# Large biomass reduction effect on the relative role of climate, fishing, and recruitment on fish population dynamics

**DOI:** 10.1038/s41598-024-59569-4

**Published:** 2024-04-18

**Authors:** Joël M. Durant, Rebecca E. Holt, Øystein Langangen

**Affiliations:** 1 Centre for Ecological and Evolutionary Synthesis (CEES), Department of Biosciences, University of Oslo, NO-0316 Oslo, Norway; 2 Centre for Environment, Fisheries, and Aquaculture Science (Cefas), Weymouth, DT4 8UB UK; 3 Section for Aquatic Biology and Toxicology (AQUA), Department of Biosciences, University of Oslo, NO-0316 Oslo, Norway

**Keywords:** Ecological modelling, Climate-change ecology

## Abstract

Many species around the world have collapsed, yet only some have recovered. A key question is what happens to populations post collapse. Traditionally, marine fish collapses are linked to overfishing, poor climate, and recruitment. We test whether the effect on biomass change from these drivers remains the same after a collapse. We used a regression model to analyse the effect of harvesting, recruitment, and climate variability on biomass change before and after a collapse across 54 marine fish populations around the world. The most salient result was the change in fishing effect that became weaker after a collapse. The change in sea temperature and recruitment effects were more variable across systems. The strongest changes were in the pelagic habitats. The resultant change in the sensitivity to external drivers indicates that whilst biomass may be rebuilt, the responses to variables known to affect stocks may have changed after a collapse. Our results show that a general model applied to many stocks provides useful insights, but that not all stocks respond similarly to a collapse calling for stock-specific models. Stocks respond to environmental drivers differently after a collapse, so caution is needed when using pre-collapse knowledge to advise on population dynamics and management.

## Introduction

The present rate of biodiversity loss^1–3^ highlights the necessity for accurate ecological forecasting and requires an in-depth understanding of the relative effect(s) of the main factors affecting change in biomass. Climate forcing^4^ and overexploitation of resources^5,6^ have often been identified as the dominant factors affecting biodiversity and abundance of marine organisms^3^. Both effects have been studied extensively in relation to fish population collapses, particularly for economically important species, thus fuelling the debate over the state of fisheries^1,6–9^. Here, we consider a collapse to have occurred when there is a large reduction in biomass, different to that of short-term natural fluctuations^10^. In the marine environment, population collapses have been attributed to overfishing^11,12^, recruitment failure^13^, and to other interacting external forces^14^ resulting in hysteresis^15,16^. Some studies argue that effective management should lead to fewer collapses due to overfishing^8^ and eventually recovery of collapsed stocks^6,17^. At the same time, mass mortality events are occurring more often globally and across taxa^18^. Such events are often caused by factors largely independent of harvesting and may or may not be responsible for collapses^19–21^.

While collapse and sometimes the proceeding recovery of the biomass level has received a great deal of attention^22^, the persisting dynamical changes caused by a large biomass reduction for fish populations are less well studied. It is well documented that the response of species to environmental factors may change over time^23,24^. In particular, a biomass collapse may lead to changes in the ecosystem^25^ and to altered population responses to environmental factors^26,27^. For example, working on long-lived fish species, Rouyer et al. found that after a collapse the stocks become more sensitive to environmental conditions, i.e., sea temperature, and less to fishing pressure^28^. They attributed this to a change in population structure; with older fish, more productive and more resistant to environmental change, having been removed from the population by fishing^28,29^. More recently, Durant et al. showed that population collapses may also alter the inter-specific interactions and response to abiotic environmental changes^30^. Despite these examples, general information on how population-level responses to environmental factors change after a collapse is scarce^22^. One reason for this limitation may be the availability of long-term, high-quality data needed to address such questions. Over the last decade, such data has been made widely available through the RAM legacy database for commercially exploited marine fishes and invertebrates^14,31^.

**Figure 1:**
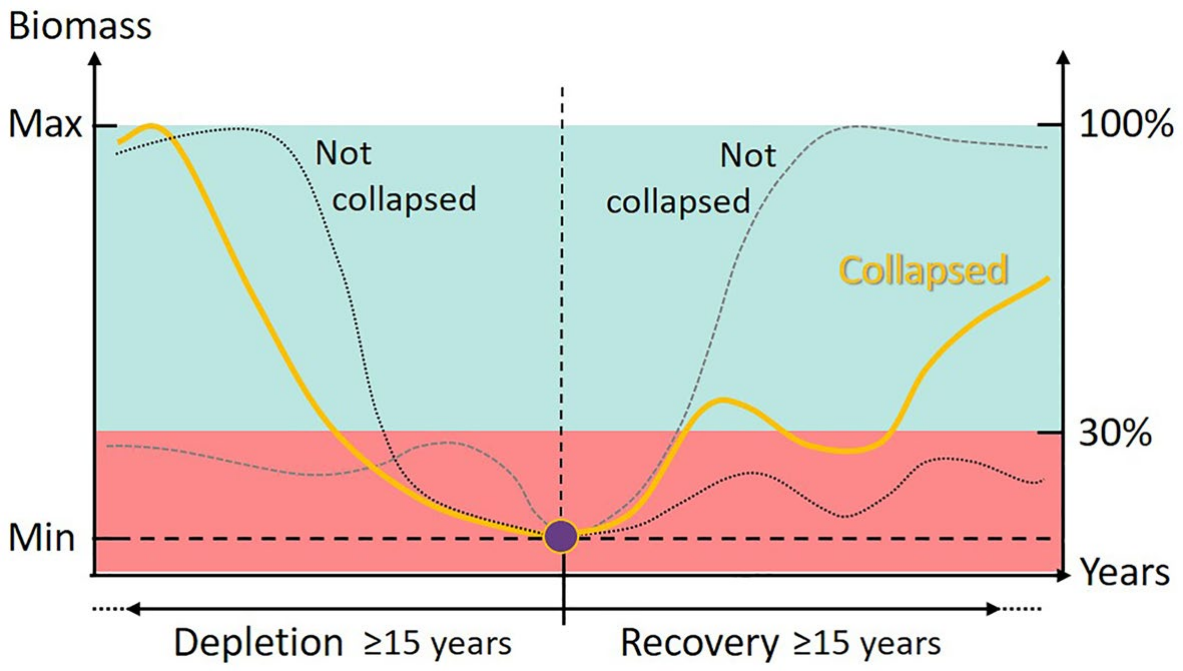
Overview of the data selection to describe a “collapsed” stock. The red area corresponds to the values lower and the green area to the values bigger than 30% of the maximum biomass recorded. The maximum biomass (100%) is found either in the period before or in the period after the lowest point (purple dot). To be retained, stocks must have both periods before and after the lowest point lasting at least 15 years. Stocks must also show a decline followed by a recovery covered by these two periods (orange curve). To do this, at least one recorded biomass for each period must be in the green area. This figure shows two examples not following these criteria and then not considered (black curves).

In this study, we investigate if biomass collapse affects a stocks response to external drivers even after some level of recovery. Our objective is to evaluate the consequences of large population biomass decline on the sensitivity to external and internal drivers in marine fishes. We then estimate the effect of fishing, environmental conditions (i.e., sea temperature), and recruitment on the change in biomass before and after a large biomass reduction of 54 recovering exploited fish stocks, representing 42 different species, from a range of habitats^32^. These three variables (fishing, temperature, and recruitment) are the common drivers documented to affect fish biomass through changes in adult and juvenile mortality (fishing^8^ and temperature^33^), reproduction (temperature^28,34^), or directly through recruitment^35^. Specifically, we work on stocks that have exhibited a biomass decrease followed by a biomass increase. We define if an increase or decrease had occurred if the biomass is reduced to at least 30% of the maximum value recorded and vice versa during the 15 year period before and after the minimum (Fig. 1 and methods), which corresponds to a 70% biomass decrease^22^.

## Methods

### Data

We extracted stock data from the RAM Legacy Stock Assessment Database (using the package *ramlegacy* in R, accessed December 2022^31^). From the 1330 stocks available in the RAM legacy database^36^, we applied selection criteria reducing the number of stocks for the analysis (the remaining number of stocks given between parentheses). We first removed all invertebrates (1144) and the fish stocks with wide ranging distributions (Atlantic, Pacific coast, North Atlantic, Southern Atlantic, and Central western pacific) (1096). From these 1096 remaining stocks, we kept only the stocks with 30 years or more biomass data (tb.data in Mt, 734), as well as with data for the variables of interest, fishing mortality (f.data in 1/year) and recruitment (r.data in numbers) (173). Finally, we wanted stocks experiencing a large decrease in biomass followed by a period of recovery (hereafter called “collapse” for simplicity), and with enough data to model the biomass change before and after this so-called collapse, i.e., with at least 15 years of biomass data. For this, we selected among the 173 stocks the ones that fitted the following criteria (see Fig. 1 and Fig. S1; 76 stocks):

i. The minimum biomass recorded allowed at least 15 years of data before and after a collapse.
ii. The biomass over time described as a V shape centred on the minimum biomass. For this, we selected stocks that have one or more values in the 15 years period before and after the minimum biomass value selected in (i) of at least 30% of the maximum biomass value recorded in the whole time series.

For the climate variable, we used sea temperature, which is arguably the most important driver of changes in the physiology and ecology of marine fish, as it has consequences at the population and ecosystem level^4,37,38^. We downloaded sea temperatures (ST in °C) from the NOAA Extended Reconstructed SST V5. ERSST-v5 is a global monthly Sea surface temperature analysis from 1854 to the present derived from ICOADS data with missing data filled in by statistical methods (https://www1.ncdc.noaa.gov/pub/data/cmb/ersst/v5/netcdf/, access December 2022). We used these estimated SST to calculate a yearly average for different geographical squares corresponding coarsely to the stock distribution (Fig. S2, Table S1).

### Modelling approach

We modelled the changes in biomass per stock as follows:

$$\Delta \left( {{\text{ln}}\left( {{\text{Biomass}}} \right)_{{{\text{yr}} - {\text{1 to yr}}}} } \right) \, = {\text{ a }} + {\text{ b}} \cdot {\text{F}}_{{{\text{yr}} - {1}}} + {\text{ c}}\cdot{\text{ST}}_{{{\text{yr}} - {1}}} + {\text{ d}} \cdot {\text{Rec}}_{{{\text{yr}}}} + \, \varepsilon_{{{\text{yr}}}} ,$$

with Δ(ln(Biomass) _yr-1 to yr_) the annual rate of change in total biomass between year *yr − 1* and *yr* calculated as ln(Biomass_yr_) − ln(Biomass_yr−1_), F the fishing mortality (instantaneous rate), ST the sea temperature, Rec the recruitment, and a, b, c and d the parameters. The method of estimating the ratios in log-scale is considered to account for temporal autocorrelation from a time series and we did not use year as a covariate^31^. We standardized the explanatory variables, F, ST, and Rec to z-scores. All estimated parameters are on the same scale; thus, their effects are directly comparable. ε_yr_ is a Gaussian distributed error term. The models were run independently for the periods before the collapse (15–20 years before the minimum value of Biomass, see above and Fig. 1 and Fig. S1) and after the collapse (15–20 years after the minimum value of Biomass).

To fit our models, we used Hamiltonian Monte Carlo methods using the Stan applied regression modelling (*rstanarm*) package in R^39^. We used the *rstanarm* default weakly informative priors. We sampled four chains for 10,000 iterations each, with 2000 post-warmup posterior iterations, with a target average acceptance probability set to 0.995 in the posterior samples. To reduce autocorrelation, we thinned the chains with a factor 10. To assess convergence, we checked that the value of the Gelman–Rubin statistic Ȓ ≤ 1.01^40^. This metric measures the ratio of the among-chain variation and the within-chain variation. Values ≥ 1.1 indicate poor mixing of the Markov Chain Monte Carlo chains and a possible model misspecification. Finally, we only retained models that had a median R^2^ ≥ 0.2 for both before and after models (Table S2; obtained using the *bayestestR* package^41^). From the original 76 collapsed stocks we modelled, the selection of a median R^2^ ≥ 0.2 retained 54 stocks. VIF analysis did not raise any serious concerns regarding collinearity^42^.

We extracted information on life history (the von Bertalanffy growth rate (K), the common length, and longevity), and the habitat (demersal, pelagic, benthopelagic, bathydemersal, bathypelagic and reef associated), from the global species database of fish species FishBase.org (using the package *rfishbase* in R, access December 2022^43^) to create broad categories for analyses (Table S3).

## Results

**Figure 2:**
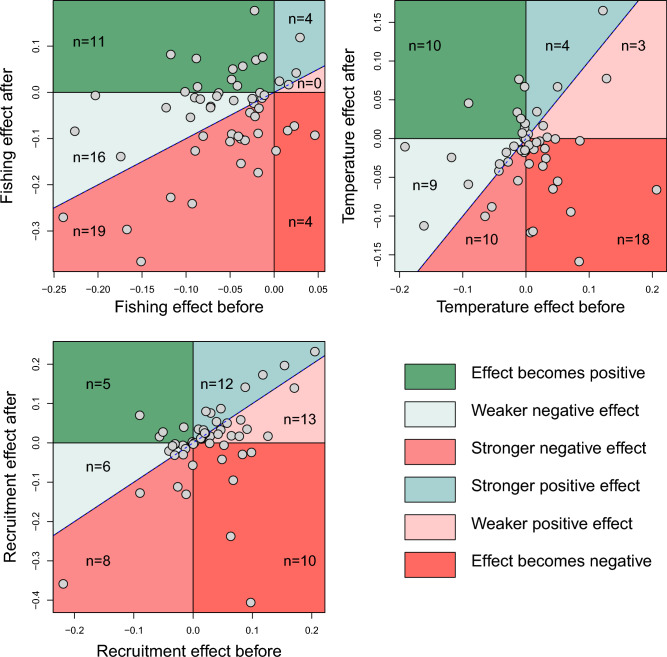
Effect of collapse on the effect strength of F, ST, and Recruitment on the change in Biomass. This figure shows the change in the effect strength for the three variables assessed in our model. For all plots, x-axis corresponds to the value of the parameters before the collapse. Y-axis corresponds to the value of the parameters after the collapse. Each dot corresponds to one stock. Stocks that exhibit no change (same effect before and after) are located on the diagonal line. Stocks that exhibit a stronger effect after the collapse than before (parameter after > parameter before, see Table S2) are located above the diagonal line and the stocks with a weaker effect (parameter after < parameter before) under the line. To group these changes, we have divided the space delimited by the parameters in six areas (area of different colours: positive effect becoming stronger, positive effect becoming weaker but remaining positive, negative effect becoming stronger, negative effect becoming weaker but remaining negative, positive effect becoming negative, or negative effect becoming positive). In each area is indicted the number of stocks included (dots) among the total of 54 stocks.

**Figure 3:**
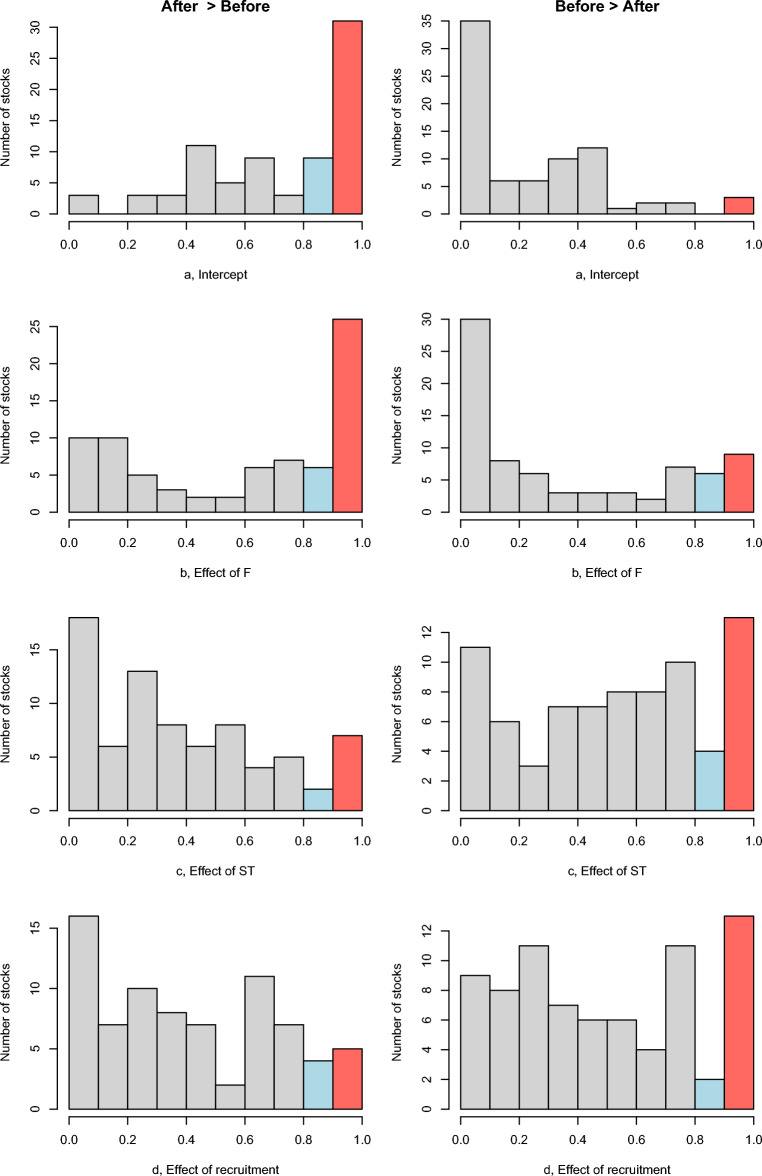
Comparison of the posterior distribution of the estimated parameters. The left column displays the number of stocks with parameters (a, b, c or d) bigger after collapse than before. The right column displays the number of stocks with parameters bigger before collapse than after. In red are stocks where 90% (0.9) of their posterior distribution is bigger than their posterior distribution of the equivalent parameter for the other model (in blue 80% bigger). The comparison was conducted by resampling 10,000 times the posteriors independently for the two models (before and after), comparing the estimates and calculating the proportion for each stock.

Our models show a varied response to external drivers post-collapse compared with pre-collapse periods. When looking at the 54 stocks together as a group, fishing had a reduced negative impact on biomass change post-collapse compared with pre-collapse (Figs. 2, 3 and Table S2). There was no strong general pattern pre- and post-collapse with respect to the effects of temperature or recruitment on biomass change. Since most stocks have a higher negative effect of F rather than a positive effect (Fig. 2), the majority of collapses seem to be associated with overfishing. At the habitat and species levels, more differences were observed between pre- and post-collapse periods. The change in the effect of sea temperature and recruitment was more variable and stronger in pelagic species compared to species associated with other habitats.

### Change in the effect of fishing (F)

**Figure 4:**
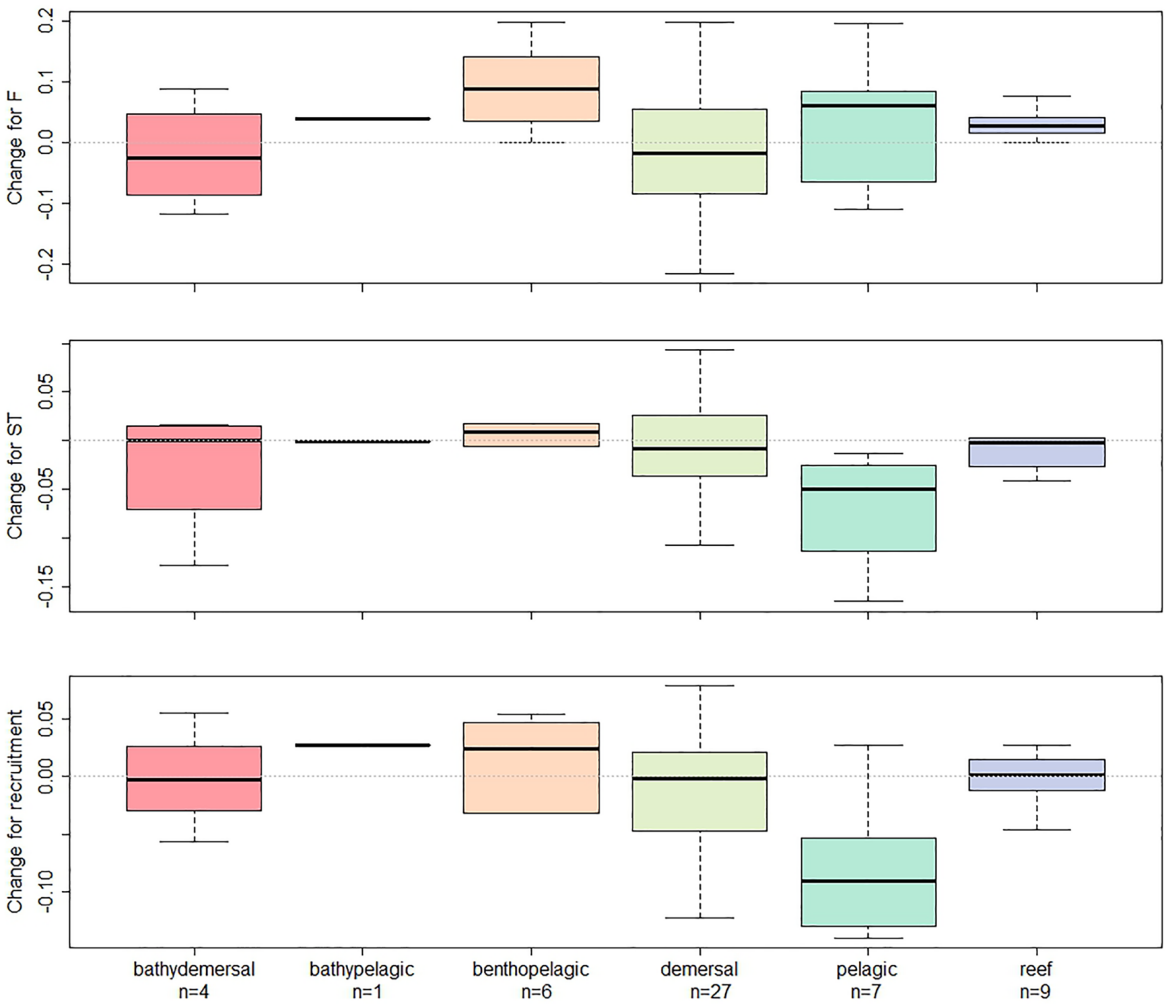
Effect of the habitat on change of effect of F, Sea temperature, and Recruitment on Biomass change after a collapse. The change of effect is the difference between the effect before and the effect after the collapse. Positive values indicate that the effect goes toward a more positive effect (see categories in Fig. 2). Conversely, a negative value indicates that the effect goes toward a more negative effect. The horizontal line is the median value. The box delimits the first and third quartiles (interquartile range (IQR) criterion) and the error bars the 5th and 95th percentiles. Figure drawn using the function *boxplot*.

The effect of F on most of the 54 stocks studied remains negative, i.e., an increase in F leads to a decrease in biomass, (with the ratio of number of stocks with a negative effect to a positive effect of 46/8 before collapse becoming 39/15 after, see Fig. 2 and Table S2). For 16 stocks the effect of F became less negative on biomass (negative parameter after > negative parameter before, i.e., the same increase of F pre-collapse led to less of a reduction of biomass increase post-collapse), for four other stocks the positive effect of F became stronger. For 19 stocks the negative effect of F became stronger, whilst for four stocks the positive effect of F pre-collapse switched to becoming negative on post-collapse biomass and 10 a negative effect of F became positive. This means that of the 54 stocks, only 23 have an increasing negative effect of F, whilst the other stocks have a reduced negative effect or even a positive one. Among the different habitats, the stronger difference is for the benthopelagic fishes with an F effect becoming strongly positive (or weakly negative) (Fig. 4, Fig. S3). The positive effect of F on fast-growing species (as indicated by K, table S3) tended to become stronger after a collapse indicating that they follow but may not affect each other (Fig. S6).

### Change in the effect of Sea temperature (ST)

The effect of sea temperature moved from being equally positive or negative to being primarily negative (with the ratio number of stocks with a negative effect to a positive effect 29/25 before collapse becoming 37/17 after, Fig. 2 and Table S2). More stocks have a lower increased biomass with increased temperature post-collapse. Nine have a negative effect of sea temperature becoming weaker after a collapse and three have a positive effect becoming weaker, four a positive effect becoming stronger, 10 a negative effect becoming stronger, whilst 18 have the positive effect of sea temperature becoming negative and 11 a negative effect becoming positive. This means that of the 54 stocks, only 28 have an increasing negative effect of ST on biomass post-collapse compared with pre-collapse, whilst 9 stocks have a reduced negative effect and 17 a positive one. Among the different habitats, the pelagic fishes show the stronger signal with an effect of sea temperature becoming negative, while the effects did not change for the other habitats (Fig. 4, Fig. S4). The positive effect of temperature on short-lived species tended to be weaker after a collapse (Fig. S6).

### Change in the effect of recruitment (Rec)

The effect of recruitment on biomass for most stocks is positive (with a ratio of number of stocks with a negative effect to a positive effect 19/35 before collapse becoming 24/30 after collapse, Fig. 2 and Table S2). However, most stocks are grouped near the diagonal line indicating weak change (Fig. 2). Six have a negative effect becoming weaker after a collapse but only 13 with a weaker positive effect becoming weaker, 12 with a positive effect becoming stronger, eight with a negative effect becoming stronger, while 10 have the effect of recruitment becoming negative and five becoming positive. This means that of the 54 stocks, only 17 have an increasing positive effect of recruitment on biomass, whilst 13 stocks have a reduced positive effect and 24 a negative one. Pelagic fishes didn’t follow the general pattern of other habitat groups, where the effect of recruitment became strongly negative post collapse (Fig. 4, Fig. S5). The negative effect of recruitment on long-lived species tended to become weaker after a collapse (Fig. S6).

## Discussion

Our results show that biomass rebuilding after a collapse often coincides with changing responses to variables known to affect population dynamics. Overall, we found by studying 54 fish populations around the world after a collapse, that the negative effect of fisheries exploitation on a population tended to be reduced whilst the effect of sea temperature and recruitment was more variable (Fig. 2). In addition, we found that this change in the effect of temperature and recruitment after a collapse were more profound in pelagic species compared to species associated with other habitats (Fig. 4, Figs. S3 and S4). The positive impact of temperature had less of an effect post-collapse for fast growing and short-lived species (Fig. S6), particularly those fishes in pelagic habitats. The resultant change of stock sensitivity to external drivers indicates that whilst biomass was rebuilt, stock dynamics may have changed in a globally different environment.

The effect of increased recruitment has, as expected, a globally positive effect on biomass (Fig. 2). However, it seems that this effect is more often negative after collapse for the seven pelagic fishes meaning that an increase in recruitment is associated to a decrease in biomass change (Fig. S5). One explanation could be that in years of high recruitment, small pelagic fish may have a higher rate of density dependent mortality, hence with a reduced total biomass increase leading to strong fluctuations in their populations. Indeed, high recruitment seems to occur when biomass is high in pelagic fish (Fig. S7). However, pelagic fishes usually show high variation in recruitment as shown for the seven pelagic stocks we analysed before but also after the collapse (Fig. S7). Note that among the seven stocks analysed, five have their recovery after 1990, hence when ST temperatures are increasing that may lead to a confounding effect (see below). Note that these small pelagic fishes are often short-lived species that might be adapted to prioritize reproduction at the cost of survival in the presence of stressors^44^.

A major mechanism that may lead to an altered population response to environmental factors after a collapse is age and size truncation (not tested in this work). For example, fisheries generally cause increased pressure on marine ecosystems^6,45^ and fishing has the unintended consequence of destabilizing the population dynamics through age-truncation^46,47^. Change of the population age structure can affect recruitment and thereby stock abundance variability^48^. However, since we only found a relatively small change in the recruitment effect (Fig. 2), the studied stocks may not have exhibited important alterations of their age-structure (in terms of truncation or restauration). Analysing 38 stocks, Goto showed that a progressive reduction in fishing pressure associated with a favourable climate may restore the maternal demographic structure (reversing age truncation) and reproductive capacity^29^. The structure of the population may thus be one of the major drivers of the collapses, acting in synergy with environmental variations and fishing pressure. In our study, fishing pressure tends to have a negative effect on biomass pre-collapse, which often becomes weaker while remaining negative or sometimes even positive post-collapse (Fig. 2). This change is consistent with the fact that management actions are often put in place after a collapse linking F more tightly to biomass. Indeed, a goal of stock management is to minimize the long-term impact of fishing. To do so management adjusts the catch, hence fishing mortality F, to a level that would lessen the effect on the stock, i.e., biomass. In addition, the effects of fishing may also be confounded with ecosystem changes such as shifts in the distribution of key resources^49^, variables that we did not take into account.

Contrary to expectations, our data-driven approach did not show a systematic global change in the effect of sea temperature (ST in our model) on fish biomass between pre- and post-collapse periods. However, the effect of temperature on biomass changes, shifting from positive to becoming negative post-collapse occurred for many pelagic fishes (Fig. 4). This change indicates that an increase in temperature before a collapse led to an increase in biomass’, whilst it led to a decrease in biomass post-collapse. One can hypothesise that since short-lived species have been highly represented in the pelagic category (Table S3) they may be more sensitive to a change in ST^50^. This did not occur for fishes within other habitats. We suggest two possible reasons for this lack of sea temperature effect. The first is that the data used in our analysis originates from an SST model from NOAA, downscaled to an area globally representing the area of distribution of the concerned stock (Table S1). Other limitations in the proxy include a coarse definition of the area (Table S1), as well as the temporally coarse average (yearly) sea temperature calculated. Thus, sea surface temperature estimates may not be spatially nor temporally optimal for all species (e.g., for demersal fishes). However, we note that by testing a spring sea temperature average we obtained similar results (results not shown).

The second reason is that sea temperature can be considered a secondary driver, in that its major influence on fish stock biomass is through growth and young survival (i.e., recruitment), as sea temperature affects earlier life stages more strongly than later life stages^24,51,52^. The relationship between sea temperature and biomass is often a two-step process whilst the other explanatory variables, fishing, and recruitment are more direct. The effects of sea temperature may be hidden by its relationship with fishing and recruitment. However, here we did not link sea temperature to the first year of life. While this choice potentially reduced the effect of sea temperature, it has the advantage of reducing the collinearity in the model notably between sea temperature and recruitment. However, only commercially exploited stocks are monitored and present in the RAM legacy database. This means that our analysis never estimated temperature and recruitment effects completely independently from fisheries exploitation thus limiting the potential scope of our results.

Finally, sea temperature changes are often proxies for more complex ocean processes affecting stocks, such as changes in circulation affecting dispersion and migration or predator–prey interactions (e.g., Refs.^53,54^) without ignoring the climate warming since 2000s (in our study most collapses occurred before the warming acceleration in the 1980s, Table S2). In addition, only commercially exploited stocks are monitored and present in the RAM legacy database.

Studying 154 marine stocks using RAM legacy data, Pinsky and Byler^14^ found that fast-growing species were more sensitive to collapse than slow-growing ones^14^. Note that in contrast to the current study, the authors excluded small pelagic fish species (families *Clupeidae* and *Engraulidae*) from their analyses. In line with this conclusion, we may expect fast-growing species to exhibit a stronger contrast before and after a collapse (stronger negative or positive effects). To investigate what determines the relative contribution of fishing, temperature, and recruitment across stocks, we looked at how the life history variables were differently distributed among the categories of change (Table S3). This showed that the fast-growing species tended to become more strongly positively affected by F (Fig. S6) similarly shown by Pinsky and Byler^14^. A reason could be that fast-growing/short lived species are less affected by fishing induced age-truncation. This line of thought is further supported by Rouyer et al., where stocks were also found to be less affected by F after a collapse^28^. The adjustment by management actions of F to the change in biomass may lead stocks to be confronted to much lower F. However, this is not what we observed for pelagic fish (Fig. S7). In addition, short-lived species tended to become less affected by sea temperature (weakly positive). Otherwise, the strength of the effect of fishing, temperature and recruitment did not change drastically with life history traits (Fig. S6).

Although the RAM legacy database provides access to data on 1330 stocks, we modelled only 76 stocks across 42 species. The reason is that the resolution and details in the data are highly variable. Previous works were also confronted to the same limitation of having data containing enough information to be modelled (154 stocks^14^, 40 stocks^19^, and 28 stocks^55^). In our case, comparing before and after collapse, we needed to have long time series. The other limitation we encountered was the relationship between sea temperature and recruitment that could have been better modelled. For instance, the sea temperature effect on recruitment may call for an interaction term. However, using such formulation would require long time series reducing our number of stocks. While possible (but see above for collinearity issues), it would require to have a model specifically tailored per stock (such as^19^). For our modelling approach, we chose a simpler model that could be applied to many species rather than a species-specific model accounting for the complexity of individual species population dynamics^56^. We also choose to use linear models for the same reason. Using simple parametric formulation has the advantage to obtain parameters for each explanatory variable and stock directly comparable. This would not have been the case with non-parametric formulation. However, this choice may explain why only 54 stocks over the 76 modelled were retained, nonlinear relationships being otherwise relatively common in fish^15,57,58^.

All things being equal, we consider that the relative simplicity of our modelling approach is counterbalanced by the number of stocks it was applied to. Consequently, we were able to quantify and compare effect sizes of environmental covariates and changes in these after a stock collapse for a range of species. We also highlight the difficulties of generalizing results when doing a metanalysis as illustrated by the variability of our results (Fig. 4).

Analysing two interacting species, Durant et al. showed that a stock collapse changes how the species respond to other species^30^. In addition, the constant change in environment can also affect trophic interactions in the system e.g., relationship between big predator fish such as cod and small pelagic fish such as herring, see^16,59^ and possibly makes the stock more susceptible to new collapses when subsequently overfished^14^. Here, our results show that after a collapse event, stocks often respond differently to environmental drivers (biotic and abiotic), calling for caution when using pre-collapse knowledge to advise on population dynamics and fisheries management post-collapse. Given our broad scale analysis and the large stock-variability observed within our results between pre- and post-collapse phases, it is difficult to draw general conclusions across all 54 stocks. As such, we suggest that detailed stock-specific studies would be better suited to inform management and increase our understanding of the ecological ramifications of collapse.

### Supplementary Information

Supplementary Information.

## Data Availability

RAM Legacy Stock Assessment Database (through the package *ramlegacy* in R, access June 2023). FishBase.org (through the package *rfishbase* in R, access August 2023). NOAA Extended Reconstructed SST V5. ERSST-v5 (https://www1.ncdc.noaa.gov/pub/data/cmb/ersst/v5/netcdf/, access December 2022).
