# Supplementary material for: Large biomass reduction effect on the relative role of climate, fishing, and recruitment on fish population dynamics
Source: Sci Rep. 2024 Apr 18;14:8995. doi: 10.1038/s41598-024-59569-4 (PMC11026439; doi:10.1038/s41598-024-59569-4)
Supplement: Supplementary file 1 — Supplementary Information. [file 41598_2024_59569_MOESM1_ESM.pdf]

Supplementary information

for

Large biomass reduction effect on the relative role of climate, fishing, and recruitment on fish population dynamics

Joël M. Durant, Rebecca E. Holt and Øystein Langangen

Figure S1: Examples of the data selection to describe a "collapse" taken in this study

Figure S2. General location of the fish stocks with collapse

Figure S3: Effect of F change on Biomass change after a collapse for 54 stocks sorted in different habitats

Figure S4: Effect of Sea temperature change on Biomass change after a collapse for 54 stocks sorted in different habitats

Figure S5: Effect of Recruitment change on Biomass change after a collapse for 54 stocks sorted in different habitats.

Figure S6. Effect of life history on the change of effect of F, Sea temperature and Recruitment on Biomass change after a collapse.

Figure S7. Changes in biomass recruitment and fishing intensity for the seven pelagic stocks modelled in the study (see Tables S2-S3). The vertical dotted lines correspond to the year of the lowest point in biomass change.

Table S1. 77 stocks from RAM legacy used for the analysis

Table S2. 77 stocks from the RAM legacy modelled for the decreasing, growing phases or both.

Table S3. Information extracted from the global species database of 42 fish species FishBase using *rfishbase* package.

Figure S1. Examples of the data selection to describe a "collapse" taken in this study (A. Northeast Arctic cod**, B.** North Sea sole**).** The red areas correspond to the values lower and the green areas to the values bigger than 30 % of the maximum biomass recorded. The maximum biomass (red dot; 100% in Fig. 1) is found either in the period before or in the period after the lowest point (purple dot and dashed line). To be retained, stocks must have both periods before and after the lowest point lasting at least 15 years. Stocks must also show a decline followed by a recovery covered by these two periods (see orange curve in Fig. 1). To do this, at least one recorded biomass for each period must be in the green area (orange dots). Note that the maximum value can sometime be outside the two periods (see red dot in A). In B, the 2006 abundance is lower than the selected minimum value since there was not enough data to model the recovery (less than 15 years). To have similar time series length, the models (before and after the minimum biomass) were all conducted on 15 to 20 years only even when more than 20 years of data were available.

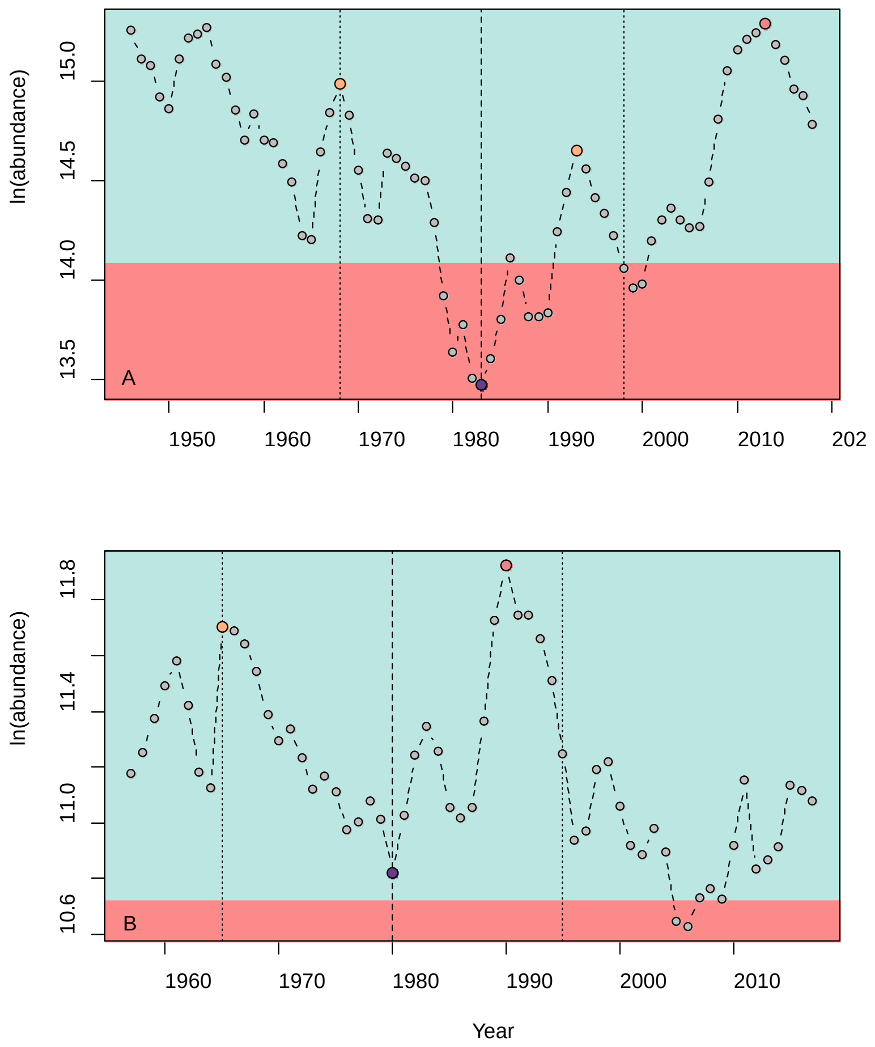

Figure S2. General location of the fish stocks with collapse (Table S1). In blue are the location of the stocks successfully modelled (Table S2). We built the map using *oceanmap* package in R (Bauer, 2020). Note that several stocks are on the same location. Most stocks in our study are from the northern hemisphere (Europe, North America) and consequently very few from the southern hemisphere (none from Africa or Asia). From the 54 retained stocks, we modelled across 42 species (Table S1-S2). Among these 42 species, 20 were demersal fishes, 6 pelagic fishes, 3 benthopelagic fishes, 4 bathydemersal fishes, 1 bathypelagic fish, and 8 reef associated fishes. More detailed can be found in Table S2.

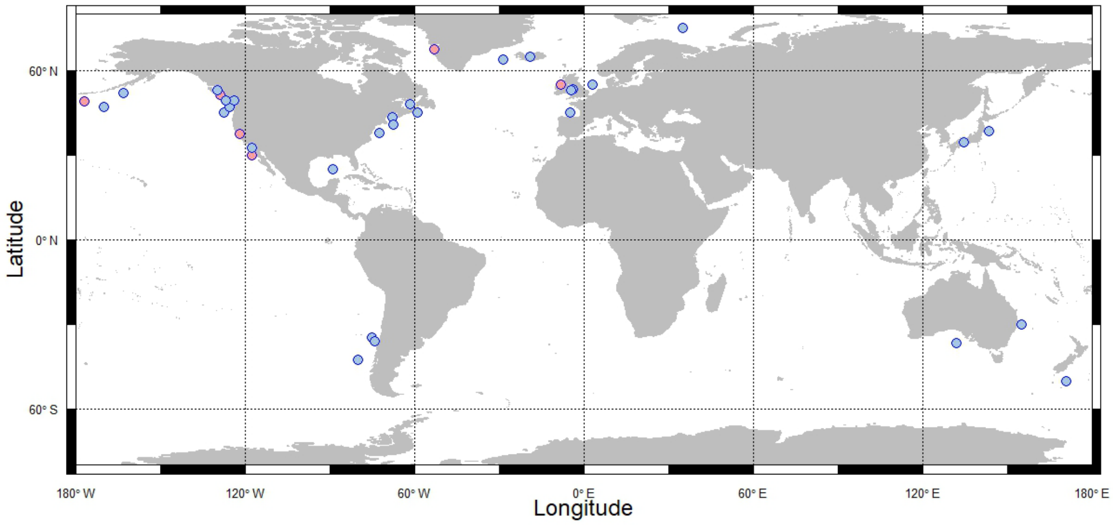

Figure S3: Effect of effect of F change on Biomass change after a collapse for 54 stocks sorted in different habitats (see categories in Fig. 2). The area of each tile is proportional to the frequency of each categorical variable both horizontal (effect) and vertical (number of stocks in each habitat). Figure drawn using the function *mosaicplot*.

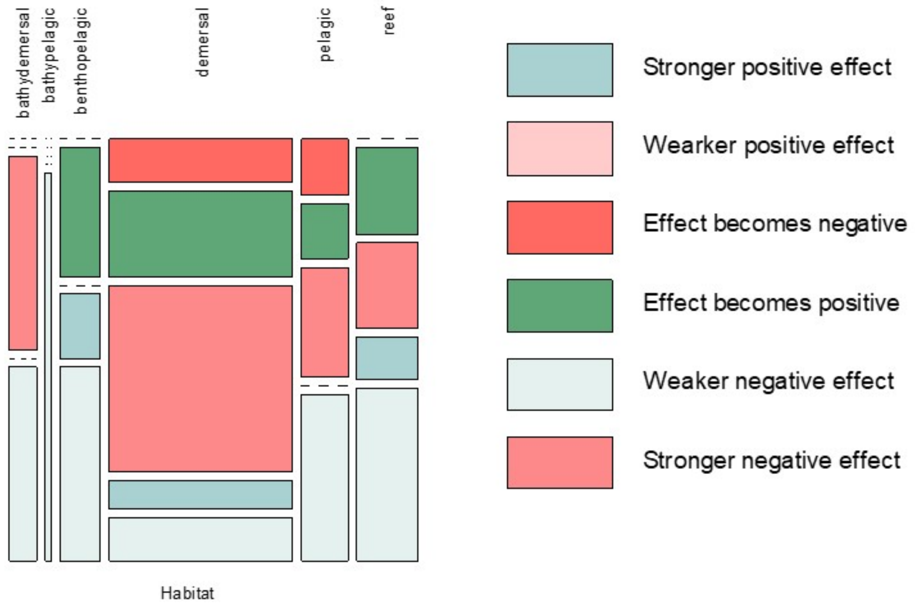

Figure S4: Effect of sea temperature change on Biomass change after a collapse for 54 stocks sorted in different habitats.

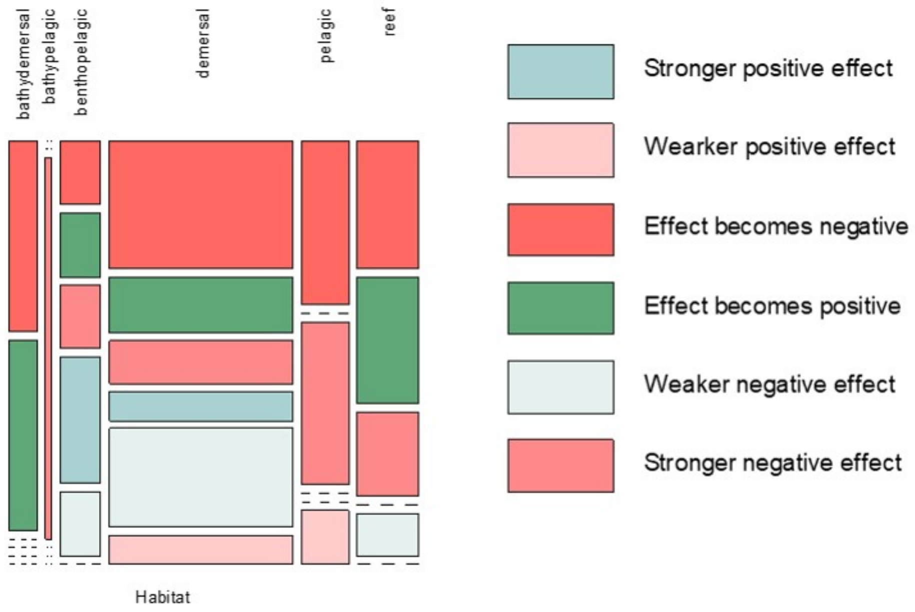

Figure S5: Effect of recruitment change on Biomass change after a collapse for 54 stocks sorted in different habitats.

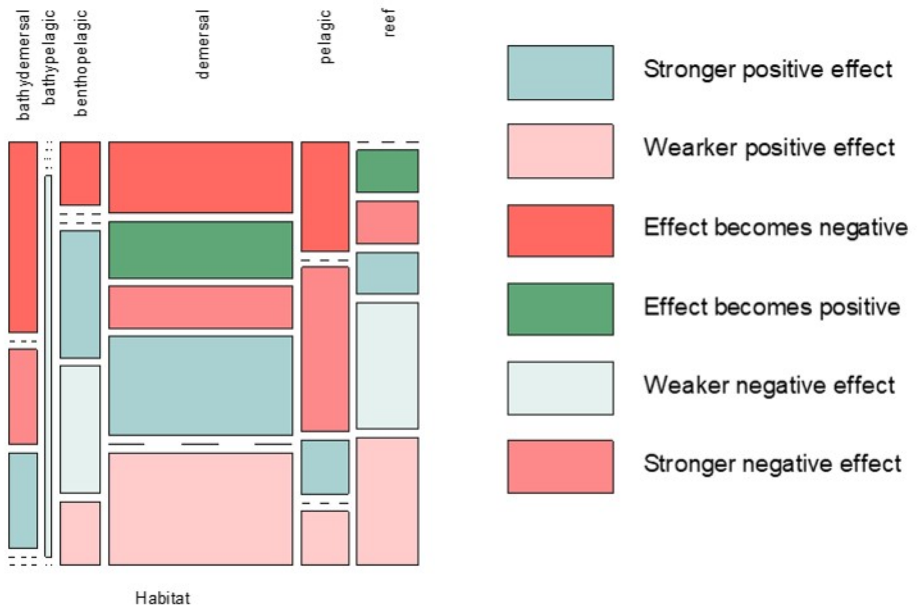

Figure S6. **Effect of life history on the change of effect of F, Sea temperature and Recruitment on Biomass change after a collapse for 54 stocks** (see categories in Fig. 2). We extracted three life history variables from the global species database of fish species FishBase.org: the von Bertalanffy growth rate (K), the common length, and longevity (Table S3).

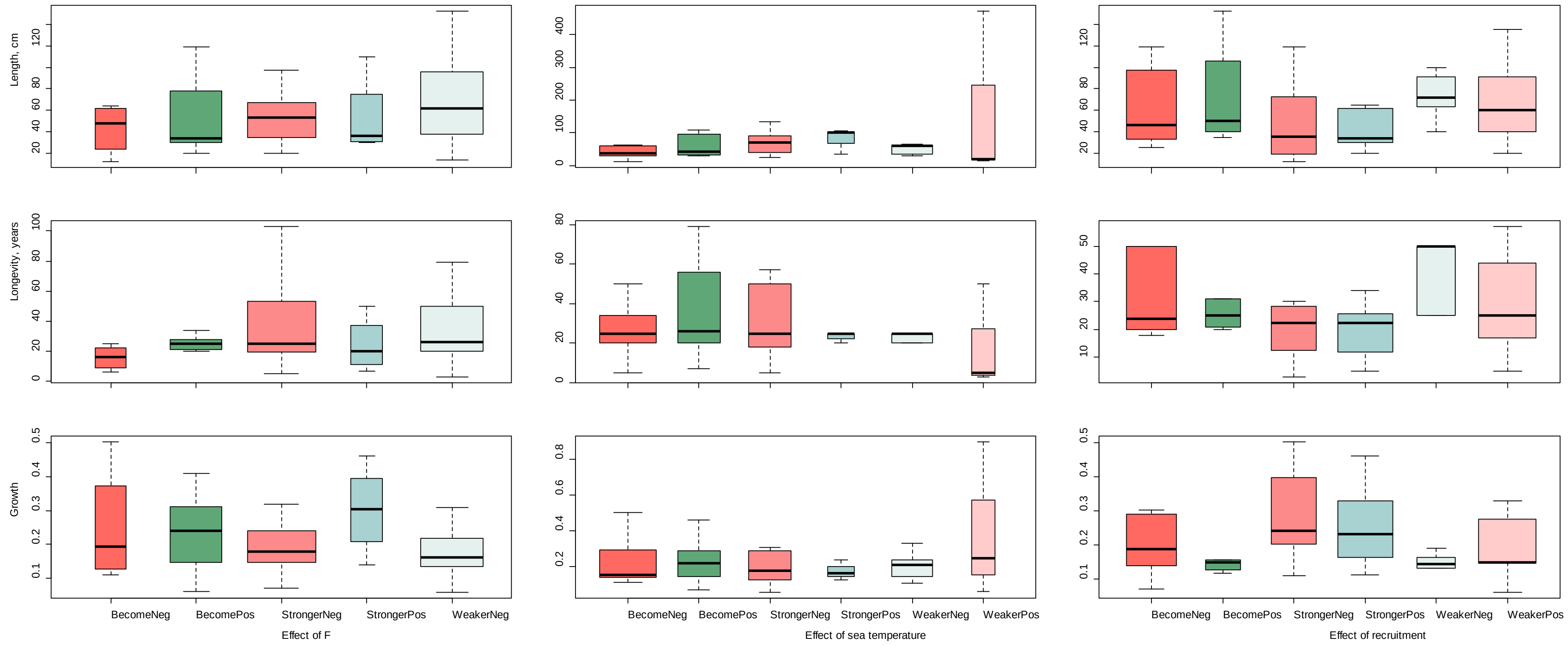

Figure S7. **Changes in biomass recruitment and fishing intensity for the seven pelagic stocks modelled in the study** (see Tables S2-S3). The vertical dotted lines correspond to the year of the lowest point in biomass change.

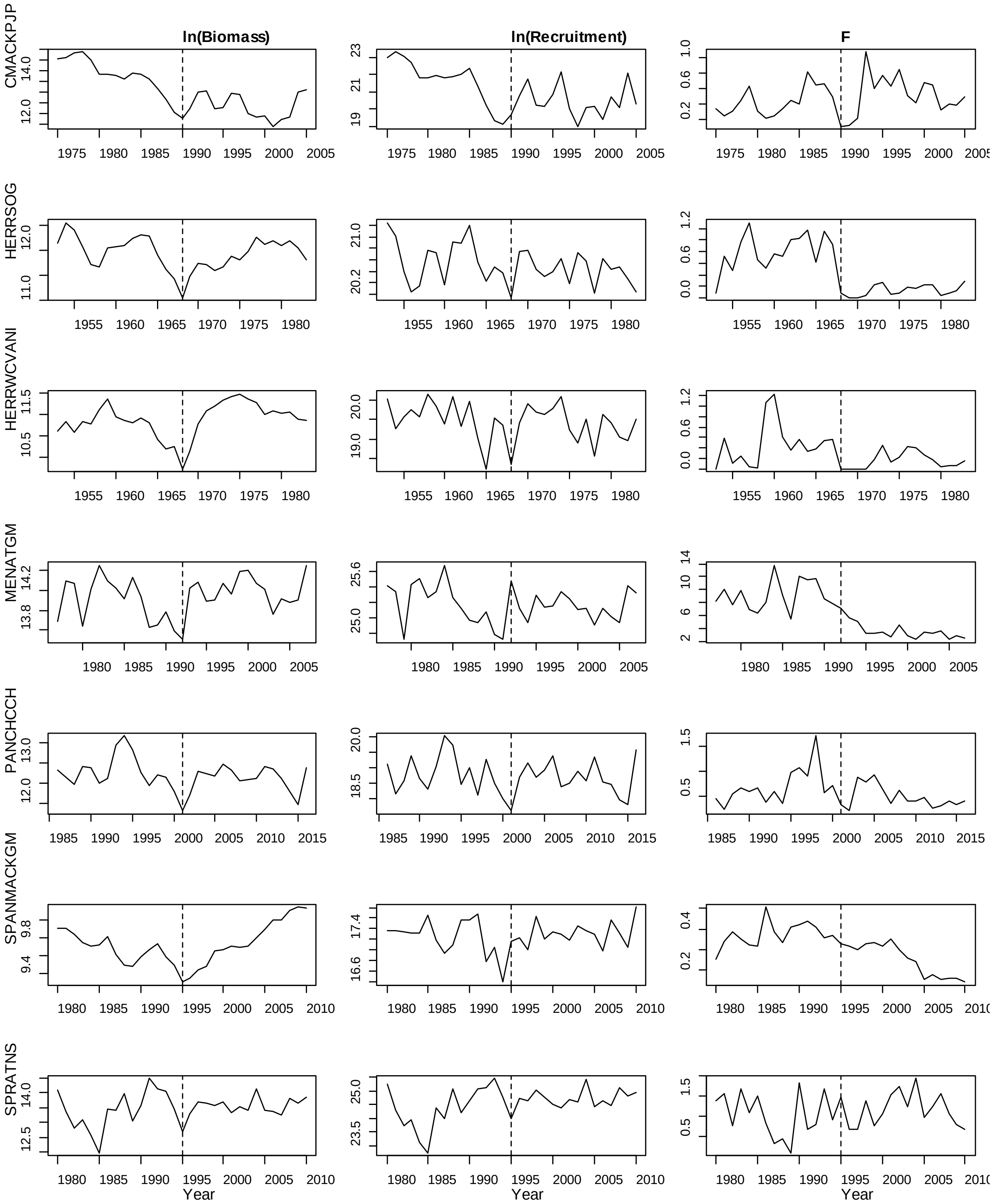

Table S1. **77 stocks from RAM legacy used for the analysis**. Latitude and longitude indicate the geographical square used to extract yearly sea temperatures from the NOAA Extended Reconstructed SST V5. The last column is the corresponding estimated surface (note that the land masses were not removed). See figure S2.

Stock ID from RAM legacy	Scientific name	Area from RAM legacy	Latitude °		Longitude °E		Surface
			South	North	West	East	000 km^2
ALSKABSAI	*Arctoraja parmifera*	Bering Sea and Aleutian Islands	41	53	194	186	809
ATHAL3NOPs4VWX5Zc	*Hippoglossus hippoglossus*	Scotian Shelf and Southern Grand Banks	43	47	307	295	420
BGRDRNSWWA	*Macruronus novaezelandiae*	New South Wales to Western Australia	-40	-33	155	109	3181
BLACKROCKCAL	*Sebastes melanops*	California	25	40	250	235	2340
BLACKROCKORECOAST	*Sebastes melanops*	Oregon Coast	41	49	235	230	350
BLACKROCKWASH	*Sebastes melanops*	Washington	46	48	236	233	51
BLUEROCKCAL	*Sebastes mystinus*	California	25	40	250	235	2340
BOCACCSPCOAST	*Sebastes paucispinis*	Southern Pacific Coast	-51	-18	290	280	3316
BRMSOJ	*Evynnis tumifrons*	Sea of Japan	27	42	138	131	1067
BSBASSMATLC	*Centropristis striata*	Mid-Atlantic Coast	33	43	295	280	1459
CALSCORPSCAL	*Scorpaena guttata*	Southern California	25	35	246	239	748
CHILISPCOAST	*Sebastes goodei*	Southern Pacific Coast	-51	-18	290	280	3316
CHROCKSPCOAST	*Sebastes nebulosus*	Southern Pacific Coast	-51	-18	290	280	3316
CMACKPJPN	*Scomber japonicus*	Pacific Coast of Japan	34	43	147	140	609
COBGM	*Rachycentron canadum*	Gulf of Mexico	20	30	278	264	1567
COD1IN	*Gadus morhua*	NAFO Subarea 1 (inshore)	60	75	317	305	850
COD3Pn4RS	*Gadus morhua*	Northern Gulf of St. Lawrence	46	50	301	296	166
CODICE	*Gadus morhua*	Iceland Grounds	62	68	348	334	438
CODNEAR	*Gadus morhua*	North-East Arctic	70	80	20	50	928
DSOLEGA	*Microstomus pacificus*	Gulf of Alaska	50	54	198	196	61
ESOLEHS	*Parophrys vetulus*	Hecate Strait	52	54	231	229	30
FMEG8c9a	*Lepidorhombus boscii*	VIIIc-IXa	43	47	358	352	210
GAGSATLC	*Mycteroperca microlepis*	Southern Atlantic coast	-60	0	10	300	44213
HAD5Y	*Melanogrammus aeglefinus*	Gulf of Maine	42	45	294	290	108

HADFAPL	*Melanogrammus aeglefinus*	Faroe Plateau	60	68	348	315	1382
HADICE	*Melanogrammus aeglefinus*	Iceland Grounds	62	68	348	334	438
HADNEAR	*Melanogrammus aeglefinus*	North-East Arctic	70	80	20	50	928
HAKENRTN	*Merluccius merluccius*	IIIa-IV-VI-VII-VIIIabd	52	58	8	-2	426
HAKESOTH	*Merluccius merluccius*	VIIIc-IXa	43	47	358	352	210
HERRCC	*Clupea pallasii*	Central Coast	35	40	240	236	196
HERRNIRS	*Clupea harengus*	Irish Sea	53	54	357	355	15
HERRNWATLC	*Clupea harengus*	Northwestern Atlantic Coast	33	43	295	280	1459
HERRPRD	*Clupea pallasii*	Prince Rupert District	52	54	231	229	30
HERRQCI	*Clupea pallasii*	Queen Charlotte Islands	51	52	232	230	15
HERRSIRS	*Clupea harengus*	ICES VIIa-g-h-j	53	54	357	355	15
HERRSOG	*Clupea pallasii*	Strait of Georgia	49	50	237	235	16
HERRVIaVIIbc	*Clupea harengus*	VIa, VIIb and VIIc	50	60	356	348	568
HERRWCVANI	*Clupea pallasii*	West Coast of Vancouver Island	48	51	235	231	97
LINGVa	*Molva molva*	Iceland Grounds	62	68	348	334	438
MEG8c9a	*Lepidorhombus whiffiagonis*	VIIIc-IXa	43	47	358	352	210
MENATGM	*Brevoortia patronus*	Gulf of Mexico	20	30	278	264	1567
MONKGOMNGB	*Lophius americanus*	Gulf of Maine / Northern Georges Bank	42	45	294	290	108
NROCKGA	*Sebastes polyspinis*	Gulf of Alaska	50	54	198	196	61
NZLINGLIN7WC	*Genypterus blacodes*	New Zealand Areas LIN 7WC-WCSI	-54	-46	175	167	509
OROUGHYSE	*Hoplostethus atlanticus*	Southeast Australia	-40	-33	155	109	3181
PANCHCCH	*Engraulis ringens*	Central Chile	-40	-30	288	284	405
PCODBS	*Gadus macrocephalus*	Bering Sea	41	53	194	186	809
PCODHS	*Gadus macrocephalus*	Hecate Strait	52	54	231	229	30
PCODWCVANI	*Gadus macrocephalus*	West Coast of Vancouver Island	48	51	235	231	97
PERCHEBSAI	*Sebastes alutus*	Eastern Bering Sea and Aleutian Islands	41	53	194	186	809
PLAICIS	*Pleuronectes platessa*	Irish Sea	53	54	357	355	227
PLAICNS	*Pleuronectes platessa*	North Sea	52	58	8	-2	15

POLL5YZ	*Pollachius virens*	Gulf of Maine / Georges Bank	42	45	294	290	426
POLLFAPL	*Pollachius virens*	Faroe Plateau	60	68	348	315	108
POLLIEG	*Pollachius virens*	Iceland Grounds	62	68	348	334	1382
POLLNEAR	*Pollachius virens*	North-East Arctic	70	80	20	50	438
POPERCHGA	*Sebastes alutus*	Gulf of Alaska	50	54	198	196	928
REXSOLEGA	*Glyptocephalus zachirus*	Gulf of Alaska	50	54	198	196	61
REYEROCKBSAI	*Sebastes aleutianus*	Bering Sea and Aleutian Islands	41	53	194	186	61
RSNAPGM	*Lutjanus campechanus*	Gulf of Mexico	20	30	278	264	809
RSNAPSATLC	*Lutjanus campechanus*	Southern Atlantic coast	-60	0	10	300	1567
SOLECS	*Solea solea*	Celtic Sea	51	55	358	353	44213
SOLENS	*Solea solea*	North Sea	52	58	8	-2	149
SOLEVIIe	*Solea solea*	Western English Channel	48	50	10	356	426
SOLEVIIIab	*Solea solea*	VIIIab	43	47	358	352	227
SPANMACKGM	*Scomberomorus maculatus*	Gulf of Mexico	20	30	278	264	210
SPHAKECH	*Merluccius gayi*	Central-Southern Chile	-55	-30	290	284	1567
SPRATNS	*Sprattus sprattus*	North Sea	52	58	8	-2	1358
SWHITSE	*Sillago flindersi*	Southeast Australia	-40	-33	155	109	426
TIGERFLATSE	*Platycephalus richardsoni*	Southeast Australia	-40	-33	155	109	3181
TILESATLC	*Lopholatilus chamaeleonticeps*	Southern Atlantic coast	-60	0	10	300	3181
WHAKEGBGOM	*Urophycis tenuis*	Gulf of Maine / Georges Bank	42	45	294	290	44213
WPOLLGA	*Gadus chalcogrammus*	Gulf of Alaska	50	54	198	196	108
YELLGB	*Myzopsetta ferruginea*	Georges Bank	40	42	295	290	61
YNOSESKACSCH	*Dipturus chilensis*	Central-Southern Chile	-55	-30	290	284	93
YSOLEBSAI	*Limanda aspera*	Bering Sea and Aleutian Islands	41	53	194	186	1358

Table S2. **77 stock from the RAM legacy modelled for the decreasing, growing phases or both**. The table presents the median of the estimated parameters following the model formulation: $a + b \cdot F_{yr-1} + c \cdot ST_{yr-1} + d \cdot Rec_{yr}$. Only 54 stocks were considered good enough (R^2 is ≥ 0.20) and used in the follow up analyses. The stocks not considered are indicated with shaded cells. In italic are model parameters (a, b, c or d) where the posterior distributions overlap by less than 20% (less by 10% if red). In bold are model parameters with a posterior distribution that are at least 80% bigger than the posterior distribution of the equivalent parameter for the other model (in red at least 90 % bigger). For example for ALSKABSAI, a(before) ≠ a(after), b(before) ≠ b(after) and c(before) ≠ c(after). 90% a(after) > a(before), 90% b(before) > b(after), and 90% c(before) > c(after). See Fig. 3.

Stock ID from RAM legacy	Data period		Data	Collapse	Before models					after models				
	start	end	length	year	a	b	c	d	R^2	a	b	c	d	R^2
ALSKABSAI	1950	2014	64	1982	*-0.02*	***-0.02***	***0.00***	0.01	0.66	***0.01***	*-0.09*	*-0.02*	0.01	0.96
ATHAL3NOPs4 VWX5Zc	1970	2010	40	1994	0.08	*-0.10*	0.03	***0.13***	0.29	0.08	***0.00***	0.02	*0.02*	0.53
BGRDRNSWWA	1960	2012	52	1981	*-0.03*	-0.01	-0.01	-0.04	0.20	**0.03**	-0.01	-0.01	-0.02	0.34
BLACKROCKCAL	1916	2014	98	1995	0.05	-0.07	0.00	0.00	0.21	0.08	-0.04	0.00	-0.01	0.15
BLACKROCKORE COAST	1892	2014	122	1999	*0.00*	-0.02	0.00	-0.02	0.94	**0.02**	-0.02	0.00	**-0.01**	0.94
BLACK ROCKWASH	1940	2014	74	1991	0.01	*-0.02*	***0.00***	0.00	0.28	0.01	***0.00***	*-0.01*	0.00	0.64
BLUEROCKCAL	1916	2007	91	1986	*-0.01*	*-0.05*	***0.01***	0.03	0.52	***0.06***	***-0.02***	*-0.01*	0.02	0.78
BOCACCSP COAST	1935	2012	77	1998	0.01	-0.07	-0.03	-0.03	0.28	0.05	-0.03	-0.03	0.00	0.26
BRMSOJ	1980	2010	30	1995	*-0.07*	-0.08	0.03	0.05	0.66	***0.05***	-0.09	0.00	0.03	0.74
BSBASSMATLC	1968	2011	43	1996	-0.03	*-0.02*	*0.00*	***0.06***	0.71	-0.01	***0.07***	***0.07***	*0.02*	0.42
CALSCORPSCAL	1916	2014	98	1976	*-0.03*	-0.04	0.01	0.00	0.18	***0.22***	-0.12	-0.04	0.00	0.22
CHILISPCOAST	1892	2014	122	1999	*0.01*	*-0.01*	-0.03	***0.00***	0.76	***0.18***	***0.20***	-0.01	*-0.05*	0.15
CHROCKSPCOAST	1901	2014	113	1998	***0.06***	*-0.10*	0.00	-0.03	0.89	*0.02*	***-0.05***	0.00	-0.03	0.77
CMACKPJPN	1970	2009	39	1990	*-0.23*	-0.24	-0.06	0.06	0.37	***0.03***	-0.27	-0.10	-0.24	0.59
COBGM	1927	2011	84	1986	-0.05	0.02	*-0.01*	***0.08***	0.84	-0.05	0.04	**0.03**	0.06	0.90
COD1IN	1976	2017	41	1998	*-0.01*	*-0.09*	***0.16***	-0.04	0.18	***0.24***	***0.11***	*-0.06*	0.01	0.34
COD3Pn4RS	1974	2009	35	1994	-0.10	-0.17	0.02	***0.10***	0.46	-0.28	-0.14	0.03	*-0.41*	0.67
CODICE	1955	2013	58	1992	*-0.06*	***0.03***	-0.04	0.03	0.21	***0.04***	*-0.05*	-0.02	0.00	0.19

CODNEAR	1946	2015	69	1983	0.09	*-0.23*	0.05	-0.03	0.32	0.10	***-0.08***	0.07	0.00	0.38
DSOLEGA	1978	2015	37	2000	0.00	-0.02	*0.00*	0.00	0.53	0.00	-0.03	***0.02***	0.00	0.93
ESOLEHS	1944	1997	53	1962	0.02	*-0.09*	***0.03***	***0.07***	0.23	0.04	***0.01***	*-0.03*	*-0.10*	0.75
FMEG8c9a	1986	2017	31	2001	0.03	*-0.09*	-0.03	0.03	0.15	0.05	***0.01***	0.02	-0.01	0.35
GAGSATLC	1962	2004	42	1997	-0.02	-0.05	0.09	*-0.02*	0.41	-0.02	0.03	0.00	***0.04***	0.20
HAD5Y	1977	2011	34	1992	*-0.07*	*-0.12*	-0.09	0.05	0.22	***0.09***	***0.08***	-0.06	-0.01	0.32
HADFAPL	1957	2017	60	1992	*-0.18*	***-0.09***	*-0.12*	0.15	0.58	**0.00**	-0.24	**-0.02**	0.20	0.54
HADICE	1979	2015	36	1997	*-0.01*	***0.05***	***0.08***	*-0.09*	0.34	***0.07***	*-0.09*	*-0.16*	***0.07***	0.23
HADNEAR	1950	2014	64	1984	0.09	-0.17	0.12	-0.09	0.38	-0.01	-0.30	0.17	-0.13	0.23
HAKENRTN	1978	2017	39	1998	*-0.02*	-0.02	0.02	*-0.02*	0.17	***0.10***	-0.02	0.01	***0.04***	0.17
HAKESOTH	1982	2017	35	1997	*-0.06*	-0.03	0.02	***0.09***	0.28	***0.05***	-0.04	0.00	*0.03*	0.50
HERRCC	1953	2007	54	1968	*0.09*	-0.16	0.03	-0.02	0.14	0.11	***0.11***	0.03	0.05	0.31
HERRNIRS	1980	2017	37	2002	*0.06*	0.03	0.03	0.17	0.72	0.05	***0.12***	-0.01	0.14	0.45
HERRNWATLC	1965	2011	46	1982	*-0.09*	*-0.02*	*-0.09*	0.03	0.28	***0.19***	***0.18***	***0.05***	0.08	0.29
HERRPRD	1953	2007	54	1968	0.00	*-0.15*	***0.13***	0.00	0.17	0.00	***-0.02***	*-0.09*	0.06	0.57
HERRQCI	1953	2007	54	1967	0.05	*-0.23*	***0.10***	-0.03	0.13	0.08	***0.06***	*-0.03*	-0.05	0.52
HERRSIRS	1958	2017	59	1978	*-0.09*	*-0.01*	-0.04	0.09	0.49	***-0.03***	***0.08***	-0.04	0.14	0.49
HERRSOG	1953	2007	54	1968	***0.15***	*-0.20*	***0.21***	***-0.03***	0.24	*-0.04*	***-0.01***	*-0.07*	*-0.11*	0.64
HERRVIaVIIbc	1957	2017	60	1979	*0.00*	-0.08	0.00	0.04	0.13	0.02	***0.00***	0.01	0.04	0.63
HERRWCVANI	1953	2007	54	1968	0.04	***-0.12***	0.00	***0.05***	0.29	0.00	*-0.23*	-0.02	*-0.04*	0.38
LINGVa	1982	2015	33	1999	*-0.01*	*-0.04*	0.00	*-0.06*	0.27	**0.08**	**0.01**	0.01	***0.02***	0.32
MEG8c9a	1986	2017	31	2002	*-0.03*	*-0.12*	***0.01***	*0.12*	0.71	***0.16***	***-0.03***	*-0.12*	***0.17***	0.43
MENATGM	1977	2011	34	1992	0.05	-0.04	0.03	0.21	0.82	0.12	0.06	-0.04	0.23	0.36
MONKGOMNGB	1980	2010	30	1996	*-0.04*	***0.00***	0.01	0.01	0.92	***0.08***	*-0.10*	0.00	0.03	0.18
NROCKGA	1961	2013	52	1976	0.00	***-0.05***	-0.03	0.01	0.50	-0.01	*-0.09*	-0.02	0.01	0.76
NZLINGLIN7WC	1972	2008	36	1993	-0.02	-0.01	0.00	**0.00**	0.42	-0.01	-0.01	0.01	-0.06	0.23
OROUGHYSE	1980	2013	33	1995	*-0.01*	*-0.07*	0.00	*-0.03*	0.94	***0.00***	***-0.03***	0.00	***-0.01***	1.00
PANCHCCH	1985	2016	31	2001	-0.02	-0.09	0.13	-0.01	0.20	0.06	-0.01	0.08	-0.13	0.24

PCODBS	1977	2015	38	1998	-0.02	*-0.09*	0.00	0.01	0.30	-0.02	***0.07***	-0.03	0.03	0.28
PCODHS	1958	2013	55	1999	**0.03**	**-0.15**	-0.05	0.08	0.54	*-0.34*	*-0.37*	-0.09	-0.03	0.64
PCODWCVANI	1956	2001	45	1985	*-0.15*	***-0.02***	-0.16	-0.01	0.50	***0.15***	*-0.17*	-0.11	-0.02	0.24
PERCHEBSAI	1960	2011	51	1981	-0.04	*-0.04*	0.00	0.00	0.35	-0.04	*-0.15*	-0.01	0.00	0.74
PLAICIS	1981	2017	36	1996	*0.00*	0.02	***0.01***	***0.10***	0.20	***0.13***	0.02	*-0.12*	*-0.02*	0.55
PLAICNS	1957	2017	60	1996	0.03	*-0.08*	-0.01	0.06	0.32	0.06	***-0.01***	-0.02	0.05	0.59
POLL5YZ	1970	2009	39	1990	*-0.04*	-0.04	0.00	0.01	0.19	***0.04***	-0.02	-0.01	0.03	0.34
POLLFAPL	1961	2015	54	1996	*-0.03*	***0.00***	-0.04	***0.08***	0.48	***0.11***	-0.13	*-0.03*	*0.02*	0.34
POLLIEG	1980	2015	35	1999	*0.00*	***-0.04***	**0.04**	0.01	0.33	***0.07***	*-0.10*	-0.07	0.01	0.33
POLLNEAR	1960	2015	55	1985	*-0.17*	*-0.02*	*-0.17*	*-0.06*	0.14	**0.06**	**0.02**	**0.04**	**-0.01**	0.45
POPERCHGA	1961	2013	52	1982	*0.01*	-0.11	0.03	***0.02***	0.19	***0.05***	***-0.04***	-0.01	*-0.01*	0.90
REXSOLEGA	1982	2008	26	2000	*-0.02*	-0.02	-0.01	-0.02	0.80	***0.05***	-0.01	***0.08***	-0.03	0.61
REYEROCKBSAI	1977	2008	31	1998	*0.00*	-0.05	-0.01	0.00	0.54	***0.04***	-0.11	0.01	0.00	0.96
RSNAPGM	1872	2011	139	1988	*-0.14*	***0.04***	*-0.01*	***0.00***	0.46	***0.14***	*-0.05*	***0.02***	*-0.02*	0.17
RSNAPSATLC	1955	2010	55	1990	*-0.12*	***-0.03***	-0.01	0.04	0.42	***0.11***	*-0.10*	-0.05	0.05	0.29
SOLECS	1971	2017	46	1997	0.01	-0.02	***0.05***	0.05	0.49	0.04	-0.05	*-0.06*	0.09	0.30
SOLENS	1957	2017	60	1980	-0.01	-0.04	0.03	0.02	0.31	0.03	-0.07	-0.03	0.07	0.13
SOLEVIIe	1969	2016	47	1993	0.01	*-0.04*	0.01	0.00	0.12	0.01	***0.01***	-0.01	-0.01	0.21
SOLEVIIIab	1984	2016	32	2001	0.02	*-0.06*	0.02	-0.01	0.14	0.01	***0.00***	0.00	0.01	0.49
SPANMACKGM	1886	2011	125	1995	0.11	-0.06	0.00	***0.04***	0.52	0.08	***0.00***	-0.01	*0.02*	0.72
SPHAKECH	1940	2013	73	1972	*-0.01*	0.00	0.00	0.06	0.22	***0.06***	0.02	-0.01	0.02	0.14
SPRATNS	1974	2015	41	1995	-0.01	0.02	0.07	-0.22	0.25	0.06	-0.07	-0.09	-0.36	0.21
SWHITSE	1947	2016	69	1997	-0.03	0.01	0.00	0.02	0.43	-0.01	0.02	0.02	0.02	0.31
TIGERFLATSE	1915	2015	100	1949	*-0.12*	-0.09	-0.02	0.02	0.54	***0.07***	-0.13	-0.01	0.03	0.42
TILESATLC	1962	2011	49	1996	*-0.05*	0.00	0.00	0.02	0.29	***0.05***	0.03	-0.02	0.01	0.15
WHAKEGBGOM	1963	2011	48	1996	*-0.06*	-0.04	-0.01	0.03	0.35	***0.05***	-0.10	0.03	0.00	0.38
WPOLLGA	1970	2014	44	2000	*-0.04*	*-0.04*	***-0.05***	-0.03	0.39	***0.09***	***0.19***	*-0.20*	-0.11	0.15
YELLGB	1973	2007	34	1988	*-0.14*	***0.02***	*-0.19*	0.02	0.23	***0.08***	*-0.08*	***-0.01***	0.08	0.44

YNOSESKACSCH	1979	2013	34	1999	*-0.01*	*-0.06*	***0.02***	*-0.05*	0.71	***0.02***	***-0.01***	*0.00*	***0.03***	0.80
YSOLEBSAI	1954	2014	60	1970	*-0.04*	*-0.05*	***0.05***	0.02	0.24	***0.06***	***0.05***	*0.00*	0.02	0.54

Table S3. Information extracted from the global species database of 42 fish species FishBase using *rfishbase* package (Boettiger et al., 2012). For the habitats of the considered species, we merged with the demersal category the bathydemersal and with pelagic category, benthopelagic, bathypelagic, pelagic-neritic and pelagic-oceanic categories. Reef category are “reef-associated” fishes. Length is the common length of the species in cm, Longevity the common longevity of the species in years, and Growth the growth coefficient K of the von Bertalanffy growth function. When FishBase gave several value for the same species, we used the median.

Scientific name	Habitat	Habitat short	Length (cm)	Longevity (year)	Growth (K)
Arctoraja parmifera	demersal	demersal	135	17	0.104
Brevoortia patronus	pelagic	pelagic	20	5	0.410
Centropristis striata	reef	reef	30	20	0.290
Clupea harengus	benthopelagic	pelagic	30	25	0.330
Clupea pallasii	pelagic	pelagic	25	19	0.290
Dipturus chilensis	demersal	demersal	152	21	0.116
Engraulis ringens	pelagic	pelagic	14	3	0.900
Evynnis tumifrons	demersal	demersal	20	5	0.250
Gadus macrocephalus	demersal	demersal	119	25	0.211
Gadus morhua	benthopelagic	pelagic	100	25	0.163
Genypterus blacodes	bathydemersal	demersal	100	30	0.108
Glyptocephalus zachirus	demersal	demersal	36	27	0.244
Hippoglossus hippoglossus	demersal	demersal	470	50	0.061
Hoplostethus atlanticus	bathypelagic	pelagic	40	149	0.060
Lepidorhombus whiffiagonis	bathydemersal	demersal	25	16	0.160
Limanda aspera	demersal	demersal	33.5	34	0.140
Lutjanus campechanus	reef	reef	60	57	0.168
Macruronus novaezelandiae	benthopelagic	pelagic	80	25	0.190

Melanogrammus aeglefinus	demersal	demersal	35	20	0.240
Merluccius merluccius	demersal	demersal	45	20	0.148
Microstomus pacificus	demersal	demersal	40	56	0.148
Molva molva	demersal	demersal	106	25	0.127
Mycteroperca microlepis	reef	reef	50	31	0.155
Myzopsetta ferruginea	demersal	demersal	64	12	0.111
Parophrys vetulus	demersal	demersal	33	22	0.295
Platycephalus richardsoni	demersal	demersal	65	12	0.222
Pleuronectes platessa	demersal	demersal	40	50	0.139
Pollachius virens	demersal	demersal	60	25	0.145
Rachycentron canadum	reef	reef	110	15	0.276
Scomber japonicus	pelagic	pelagic	30	18	0.303
Scomberomorus maculatus	pelagic	pelagic	91	5	0.310
Sebastes aleutianus	bathydemersal	demersal	97	205	0.070
Sebastes alutus	bathydemersal	demersal	53	103	0.130
Sebastes melanops	reef	reef	63	50	0.143
Sebastes mystinus	reef	reef	61	44	0.149
Sebastes nebulosus	reef	reef	45	79	0.193
Sebastes paucispinis	reef	reef	91	50	0.130
Sebastes polyspinis	demersal	demersal	41	57	0.180
Sillago flindersi	demersal	demersal	32	7	0.460
Solea solea	demersal	demersal	35	26.4	0.320
Sprattus sprattus	pelagic	pelagic	12	6	0.503
Urophycis tenuis	demersal	demersal	70	23	0.162

References

Bauer, R. K. 2020. oceanmap: A plotting Toolbox for 2D Oceanographic Data. R package version 0.1.1 edn. https://CRAN.R-project.org/package=oceanmap.

Boettiger, C., Lang, D. T., and Wainwright, P. C. 2012. rfishbase: exploring, manipulating and visualizing FishBase data from R. Journal of Fish Biology, 81: 2030-2039.
